# Health risks of airplane boarding methods with apron buses when some passengers disregard safe social distancing

**DOI:** 10.1371/journal.pone.0271544

**Published:** 2022-08-01

**Authors:** R. John Milne, Liviu-Adrian Cotfas, Camelia Delcea, Liliana Crăciun, Anca Gabriela Molănescu

**Affiliations:** 1 David D. Reh School of Business, Clarkson University, Potsdam, NY, United States of America; 2 Department of Economic Informatics and Cybernetics, Bucharest University of Economic Studies, Bucharest, Romania; 3 Department of Economics and Economic Policies, Bucharest University of Economic Studies, Bucharest, Romania; Central Queensland University - Gladstone Campus, AUSTRALIA

## Abstract

Many airlines instituted social distancing practices to keep passengers safe during the pandemic. The practices include keeping the middle seats empty, reducing the number of passengers taking an apron bus from the terminal to the airplane, and prescribing that passengers maintain 1 m social distance of separation from other passengers in the aisle while advancing to their seats. However, not all passengers comply with a prescribed 1 m aisle social distance. Through agent-based simulations of passenger boarding when apron buses are used, we examine boarding policies adapted for the pandemic when the level of passenger compliance varies. To compare policies, we consider the duration of time that passengers are too close to other passengers while walking or standing in the aisle. We consider other health metrics from previous research and the time to complete boarding of the airplane. We find that the WilMA–Spread and Reverse-pyramid–Spread boarding methods provide favorable outcomes. Airlines should use WilMA–Spread if their primary concern is the risk to passengers while walking down the aisle and Reverse-pyramid–Spread if they want faster times to complete boarding of the airplane and reduced risk to aisle seat passengers from later boarding passengers. The level of the passengers’ non-compliance with the prescribed aisle social distance can impact a health metric by up to 6.75%—depending on the boarding method and metric. However, non-compliance reduces the time to complete boarding of the airplane by up to 38.8% even though it increases the average time an individual passenger spends boarding.

## Introduction

The onset of the novel coronavirus led to changes in many aspects of commercial air travel [1]. Literature is emerging on how to minimize the health risks associated with the airplane boarding process [2–4], including evaluating boarding methods adapted for the pandemic [5, 6], and modelling the passengers’ deplaning process [7, 8]. Related work analyzes the social and emotional perspectives of airline passengers [9] including their willingness to fly [10] and travel behaviors in pandemic times [11]. Broader issues concern long-term planning [12] and other strategic responses of the airlines to the pandemic [13, 14], the governments’ willingness to support airlines [15], the impact of the pandemic on transport markets [16], on airline industry [17] and on airline stock prices [18], the prospects for global recovery [19–22], and even the reconsideration of the entire foundation of the global aviation system [23]. Sun et al. [24] provide a comprehensive review related to air transportation during the COVID-19 pandemic, while Riquelme et al. [25] discuss the contagion modeling and simulation in transport and air travel networks in the context of COVID-19 pandemic.

Airplane boarding methods used before COVID-19 have been modified to accommodate the new social distancing rules imposed by the pandemic and tested for performance [6]. New methods have been proposed and tested by taking into account the social distance and a wide range of interacting variables, such as passengers’ movement characteristics, the occurrence/absence of seat and aisle interferences, the possibility to board or not the passengers one-by-one, the presence of groups of passengers travelling together, the percentage of passengers carrying hand luggage aboard the airplane, the size of the hand luggage, the airplane characteristics, whether the passengers use a jet bridge or apron buses to board from the airport terminal, and more [2, 5, 24, 26].

However, most of the studies assume a full passenger load and that passengers leave the airport terminal to enter the airplane through a jet bridge [2, 27–32]. Only one recent study assumes passengers take apron buses from the terminal to the airplane during the pandemic [5]. This may partially stem from difficulties in using some boarding methods (especially the ones “by seat”) with apron buses [33, 34]. During the pandemic, apron buses continue to be used at many airports [35, 36] but their capacity is reduced. A recent announcement by the COBUS Industries GmbH on their Facebook and Twitter pages states that their apron bus model COBUS 2700s, which normally accommodates 77 passengers, can only transport 10 passengers when the prescribed social distance restrictions are respected [37].

Meanwhile, keeping the social distance inside the airplane and respecting the recommendations of the International Air Transport Association (IATA) of 1–2 m between passengers is burdensome as it reduces the number of available seats [6, 38]. As a result, most of the recent literature considers a milder approach, inspired by the recommendations made by the World Health Organization (WHO) and by many airlines’ boarding practice, namely an aisle social distance of 1 m—the minimum distance between adjacent passengers walking or standing in the aisle—and leaving the middle seats empty on airplanes with a narrow body and three seats on each side of the aisle [3, 24, 39–42].

A recent review paper on the COVID-19 pandemic and air transportation pointed out that the passengers’ willingness to comply with the prescribed aisle social distance may impact the boarding result; however, those authors and the papers they cite do not quantify that impact [24]. Other research indicates that passengers’ non-compliance with other rules while boarding an airplane–especially manifested by the passengers’ late arrival to the boarding gate–can impact on the overall performance of the boarding method [30, 43–45]. Related research indicates that the passengers’ delays might affect the entire transportation process by producing a cascading effect [46]. Researchers have also observed non-compliance with social distancing rules in other contexts [47, 48].

In this paper, we analyze the variation in health risks associated with the airplane boarding process in pandemic times when the transport of the passengers to the airplane is made through the use of apron buses and when some of the passengers disregard the rule for maintaining a minimum of 1 m of aisle social distance. In this regard, while the risks cannot be completely eliminated, they may be reduced by improving protection measures for known and potential risks. The paper proposes and analyzes several boarding methods adapted for the pandemic situation when the boarding is made through both the front and rear doors of the airplane and the use of apron buses with a pandemic capacity (due to social distancing) of 10 passengers per bus trip from the airport terminal to the airplane. To observe how the health metrics and the operational performance metrics change as a result of varying levels of the passengers’ non-compliance to the prescribed aisle social distance of 1 m, an agent-based model is created and used for stochastic simulations. Our paper is unique in considering the impact of varying degrees of non-compliance with aisle social distance restrictions.

The boarding methods presented in the following are inspired by some of the “by group” methods from the scientific literature, namely Back-to-front, WilMA, and Reverse-pyramid. These are the most common boarding methods in the research literature and airplane boarding practice [24, 49] and have proven over time to provide some of the best boarding times and health metrics, while having simple rules of forming the passengers’ groups. In the context of our paper, a boarding group consists of the set of passengers taking a particular trip on an apron bus, that is, the first trip, second trip, etc. The passengers selected to board the first apron bus trip are the first to enter the airplane.

The general rules associated with the Back-to-front method divide the passengers into boarding groups based on their seats, stating from the back of the airplane. Usually, about five boarding groups are used when the airplane is connected to the airport terminal with a jet bridge and the passengers board through the airplane’s front door [50]. The group of the passengers having seats closest to the rear of the airplane is called to board first, while the last group to board have seats near the front part of the airplane [32, 51, 52]. The boarding sequence of the passengers within a group is random. This practice of random boarding of passengers within the same group applies to all the boarding methods.

The WilMA (Windows, Middle, Aisle) boarding method, also called Outside-in, divides the passengers into three groups based on their seats’ location: adjacent to the window, middle, or adjacent to the aisle. The first group called for boarding contains the passengers with window seats, followed by the group with middle seats, and finally the group with aisle seats [53, 54].

The Reverse-pyramid boarding method follows a diagonal scheme for boarding the passengers. While most of the groups formed in the Reverse-pyramid scheme load diagonally, the first group of passengers sit in the window seats nearest the back of the airplane and the last groups of passengers to board sit in aisle seats near the front of the airplane. This method, developed by van den Briel et al. [55], provide characteristics that makes it safe for boarding in the context of pandemics as it minimizes the interaction among the passengers [40]. Compared to Back-to-front, both WilMA and Reverse-pyramid methods have zero seat interferences as none of the passengers with a window or middle seat need to ask previously seated passengers with a middle or aisle seat closer to the aisle to clear the way to their assigned seats.

The three methods are adapted for the social distancing conditions imposed by the occurrence of the novel coronavirus, in which the seat social distance is preserved by keeping the middle seat empty. With this condition, an airbus A320 airplane, with 30 rows of seats and two aisle seats and two window seats in each row, will have 120 occupied seats when fully loaded [3]. In our study, passengers are transported between the airport terminal and the airplane through apron buses that can accommodate 10 passengers per trip with social distancing during a pandemic. Upon arriving at the airport, the 10 passengers depart from the apron bus in a random sequence. We assume that passengers are assigned to apron buses (i.e. to boarding groups) in a way that 5 passengers of each group, those having seats in rows 1–15, will enter the airplane through its front door, while the 5 passengers of each group, those having seats in rows 16–30, will enter the airplane through its rear door. We assume that all 120 seats are occupied, resulting in 12 apron bus trips (i.e. 12 boarding groups) required to transport all passengers. The applicability in practice of the proposed schemes is simple as it only requires the airline to stamp on the boarding tickets the number of the apron bus to which a passenger is assigned to and whether the passenger should use the airplane’s front or rear door. The airplane boarding schemes are symmetrical with respect to the middle of the airplane. We describe the methods adapted for this context in further detail below.

### Back-to-front

**Fig 1:**
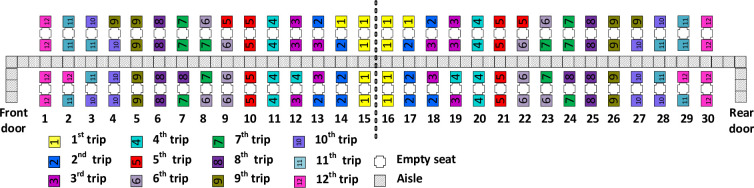
Back-to-front boarding method.

The Back-to-front method for airplane boarding, with apron buses and the middle seat empty, involves passengers boarding first when their seats are located nearest to the middle of the airplane. When we refer to “back” in this paper, we are referring to the row that is positioned in the middle of the airplane, at the longest distance with respect to each of the two entrances, namely row 15 in the case of the front (left in the Fig 1) half of the airplane and row 16 for the rear (right in Fig 1) half of the airplane. The end of the two halves of the airplane is marked in the schemes by a dotted vertical line.

As illustrated in Fig 1, with Back-to-front boarding, the first group (i.e. the first apron bus trip) of passengers to board are those with seats closest to the back. The second group to board (i.e. the second bus trip) are those with seats nearly as close to the back, and so forth, until the 12^th^ and final bus trip contains the 10 passengers with seats closest to an airplane door. When there are seats that are equally close to the back, our implementation of the Back-to-front method favors the assignment of window seat passengers to the earlier apron bus trip.

### WilMA—Back-to-front

**Fig 2:**
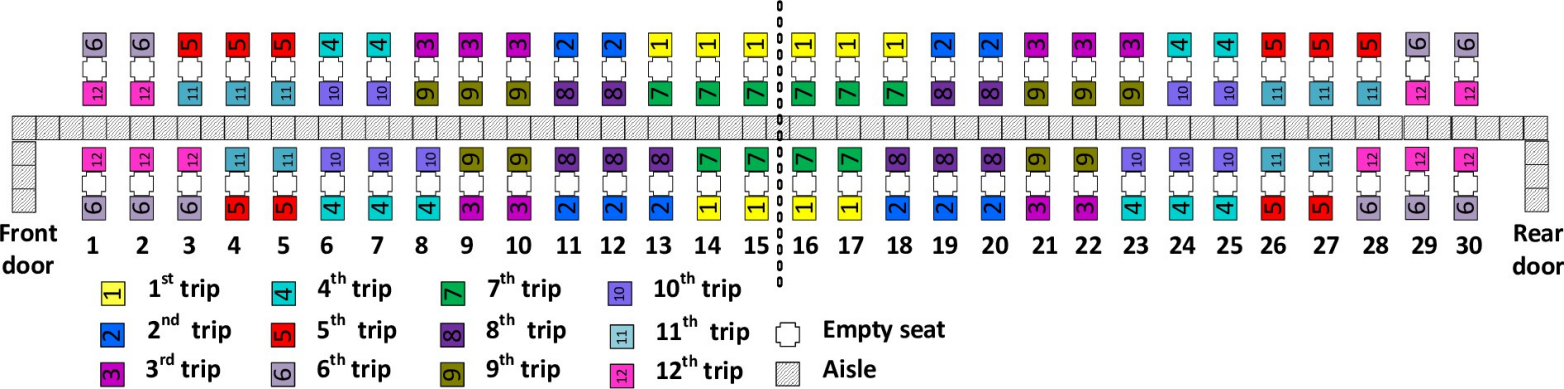
WilMA—Back-to-front boarding method.

With WilMA, the window seat passengers board the airplane before the aisle seat passengers. The WilMA–Back-to-front boarding method applies the WilMA principle first and foremost, and given a WilMA boarding sequence, assigns passengers to apron bus trips with the sequence that is as much Back-to-front as possible. This scheme is provided in Fig 2. Observe that the first apron bus trip consists of the window seat passengers seated closest to the middle of the airplane, and the 12^th^ and final apron bus trip consists of passengers with aisle seats closest to an airplane door. The first six apron bus trips consist of window seat passengers and the final six apron bus trips consist of aisle seat passengers. Within each of those two sets of apron bus trips, the sequence is Back-to-front.

### WilMA—Spread

**Fig 3:**
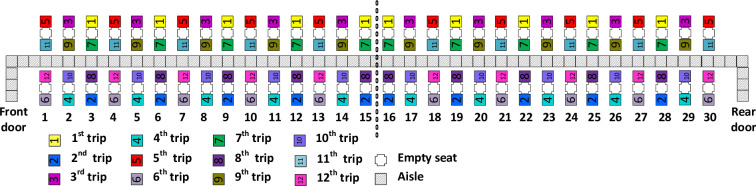
WilMA—Spread boarding method.

The WilMA–Spread method applies the WilMA principle first and foremost, and given the WilMA boarding sequence, assigns passengers to bus trips so that their seats in each bus are spread throughout the airplane’s rows as much as possible as illustrated in Fig 3. Observe that each passenger in a boarding group (bus trip) is separated from other passengers of the same group by three rows in its half of the airplane. We refer to this relatively large separation between seated passengers as “spread.”

### WilMA–Back-to-front–one-per-row

**Fig 4:**
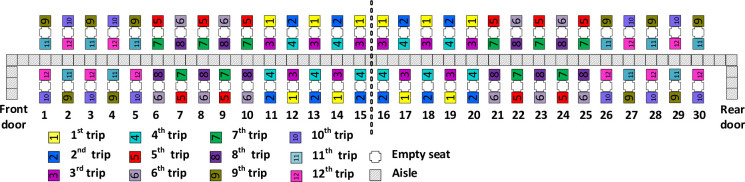
WilMA—Back-to-front boarding method.

Like WilMA–Back-to-front, the WilMA–Back-to-front–one-per-row method applies WilMA as the top priority and Back-to-front as the second priority, with the only difference being that the latter method limits to one the maximum number of passengers from a row to be boarded on any given bus trip. The WilMA–Back-to-front–one-per-row method is illustrated in Fig 4. In some respects, this method is a compromise between the two earlier WilMA methods due to it having more spread than WilMA–Back-to-front and less spread than WilMA–Spread.

### Reverse-pyramid–Steep

**Fig 5:**
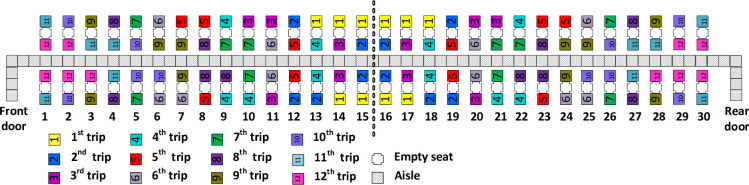
Reverse-pyramid—Steep boarding method.

In the Reverse-pyramid–Steep method, the first bus trip (i.e. first boarding group) of passengers have the window seats closest to the middle of the airplane, and the 12^th^ and final bus trip of passengers have aisle seats closest to a door of the airplane. For each of the first 5 intermediate groups (groups 2 to 6), there are 2 aisle seat passengers and 3 window seat passengers. For each of the final 5 intermediate groups (groups 7 to 11), there are 3 aisle seat passengers and 2 window seat passengers. Based on its boarding rules, with Reverse-pyramid–Steep, the window seat passengers of each succeeding group sit as close to the middle of the airplane as possible (Fig 5).

### Reverse-pyramid–Spread

**Fig 6:**
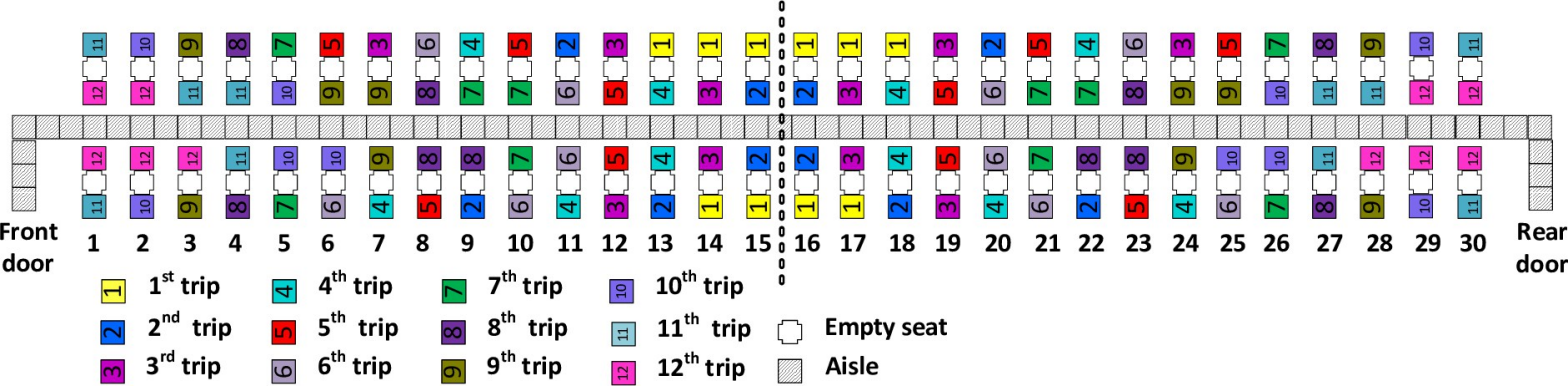
Reverse-pyramid–Spread boarding method.

The Reverse-pyramid–Spread method has the same assignments of passengers to bus trips as done with the Reverse-pyramid–Steep method, with one exception. That one difference pertains to the window seat passengers assigned to the first 5 intermediate groups (groups 2 to 6) as illustrated in Fig 6. With Reverse-pyramid–Spread, the window seat passengers of each of the first 5 intermediate groups have more rows of separation between them (i.e. more spread) than with the Reverse-pyramid–Steep method.

The remainder of the paper is structured as follows. The following section describes the metrics and scenarios used in the simulation process. The subsequent section describes the key characteristics of the agent-based model, while the next major section discusses the numerical results of the simulations. The paper closes with conclusions.

## Metrics and scenarios

For evaluating the performance of the boarding methods, a series of metrics is used along with scenarios in which the compliance of the passengers to the prescribed aisle social distance is varied.

### Metrics

Our focus in this paper is on passenger health when passengers’ compliance is imperfect with respect to aisle social distance during pandemic times when apron buses are used. Consequently, most of the metrics for evaluating the boarding methods pertain to health and one operational metric is used, namely the average time to complete boarding of the airplane.

Three of the five metrics related to passenger health are: aisle seat risk, window seat risk, the total number of seat interferences [3, 5, 41]. The other two health metrics—aisle standing risk and the individual boarding time—were created to capture health risks when there is an endemic (or an major health situation) that is unbeknownst to the traveling public and thus no passengers practice aisle social distancing [56].

**Aisle seat risk** measures the overall risk experienced by seated passengers with aisle seats due to a possible exposure to infectious passengers who walk by them to their assigned seats. The aisle seat risk is measured in seconds, a prolonged exposure being connected to a greater health risk for the previously seated passengers with aisle seats. The formula for calculating the aisle seat risk is given in the following [3, 5, 41]:

$$AisleSeatRisk=\sum_{p}\sum_{r\leq RowSit_{p}}\left(RowTime_{pr}*\sum_{p^{\prime }<p}AisleSeat_{p^{\prime }r}\right)$$

where

*p* = passenger advancing towards his or her seat

*r* = row index

*RowSit*_*p*_ = row in which passenger *p* has an assigned seat

*RowTime*_*pr*_ = time that passenger *p* spends in row *r*

(this duration begins when passenger *p* begins to enter row *r*

and concludes when passenger *p* begins to leave row *r*;

this convention is chosen because a passenger’s nose and mouth are at the front of the passenger)

*p’* = passenger boarding before passenger *p*

*AisleSeat*_*p’r*_ = 1 if passenger *p’* has an aisle seat in row *r*

= 0 otherwise

**Window seat risk** measures a risk that is similar to aisle seat risk, with the only difference being that it is calculated for seated passengers with window seats that are potentially exposed to passengers walking by them to their assigned seats. The risk is measured in seconds by using the formula [3, 5, 41]:

$$WindowSeatRisk=\sum_{p}\sum_{r\leq RowSit_{p}}\left(RowTime_{pr}*\sum_{p^{\prime }<p}WindowSeat_{p^{\prime }r}\right)$$

where

*WindowSeat*_*p’r*_ = 1 if passenger *p’* has a window seat in row *r*

= 0 otherwise

**Fig 7:**
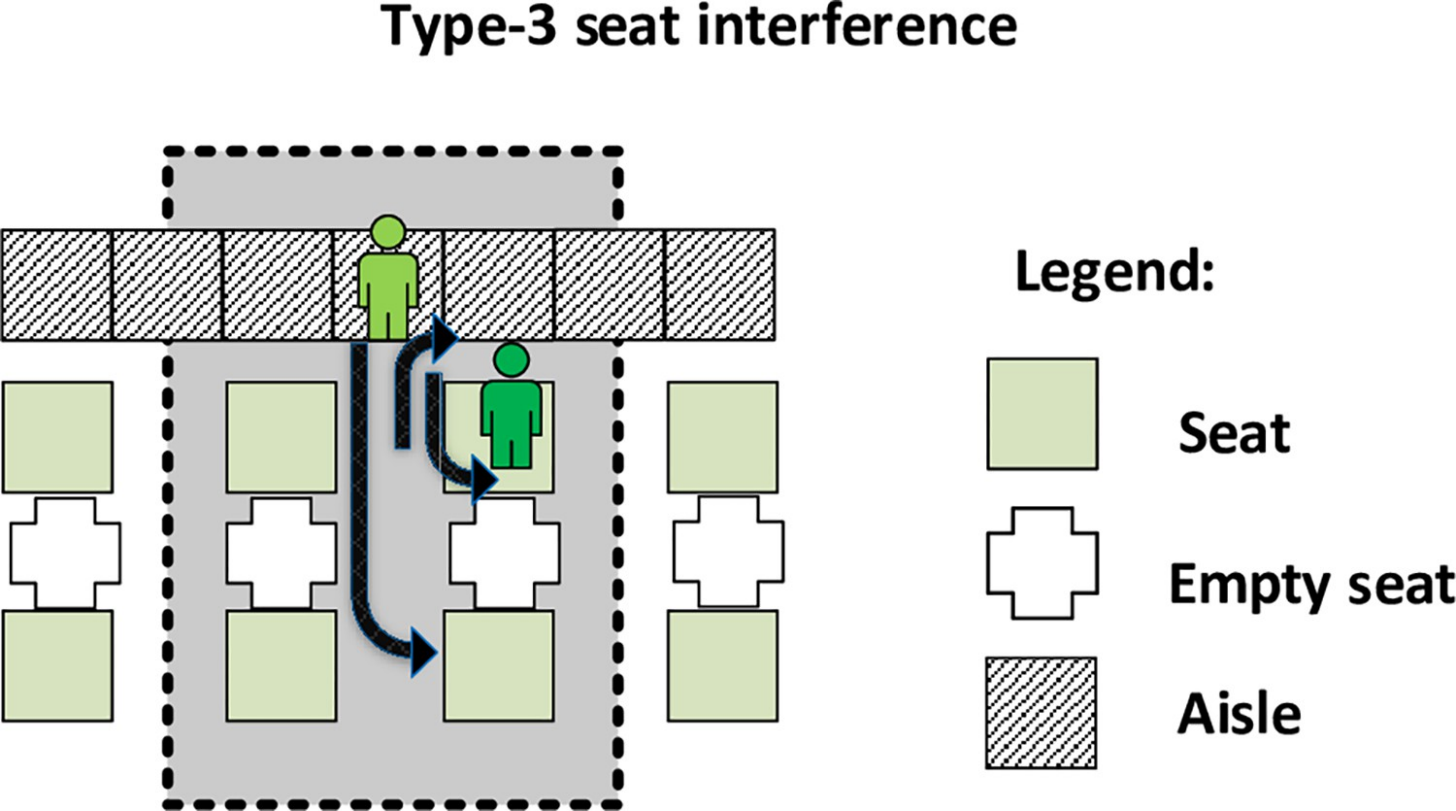
Type-3 seat interference.

**Total number of seat interferences** refers to the number of the seat interferences recorded for the entire boarding process. A seat interference occurs each time a passenger having a seat in a particular row needs to ask the other passengers already seated in the same row on the same side of the airplane to stand up to clear the later boarding passenger’s path to his or her assigned seat. In the research literature, 4 types of seat interferences (known as type-1, type-2, type-3 and type-4) are acknowledged for a narrow body airplane with three seats on each side of the aisle [57]. Due to the prescribed social distance among seats in times of pandemics, in which the middle seats are empty, the only type of seat interferences that might be encountered in airplane boarding is the type-3 seat interference. The type-3 seat interference occurs when a passenger with a window seat arrives near its allocated seat after the passenger with the aisle seat located in the same row and the same side of the airplane has already occupied his/her seat. As a result, the passenger with the aisle seat, needs to clear the way of the passenger with the window seat, which increases the health risk of both passengers due to the possible contagion as the two passengers may get close to each other when crossing. Fig 7 briefly presents the scheme of this situation–the passenger in light-green arrives near his or her allocated window seat after the passenger in dark-green has already taken his or her assigned seat in the same row. The dark-green passenger needs to leave the seat, then the light-green passenger sits in the window seat, and finally the dark-green passenger returns to sit in the aisle seat.

**Aisle standing risk** measures the total weighted duration of time–summed over all passengers–that the passenger stands or walks in the aisle when there is less than 1 m of distance between the passenger and any previously boarding passenger in the aisle in front of him or her. This metric assumes that 1 m of social distance is safe and less than 1 m of social distance is not as safe. We weight more heavily those distances that are shorter than 1 m by a larger amount. We apply weights that are equal to the amount of distance shorter than 1 m of the two adjacent passengers walking or standing in the aisle. With this assumption, passengers that are separated by 0.6 m (which is a separation of 0.4 m less than the 1 m minimum safe target), would be weighted as twice as influential, that is twice as bad, as if they were separated by 0.2 m less than 1 m, that is, separated by 0.8 m, (because 0.4 is twice the value of 0.2 = 1 m– 0.8 m) and 33% less influential than if the they were separated by 0.4 m (because 1 m– 0.6 m = 0.4 m which has a weight of 0.4 which is 33% less than the weight of 0.6, which is the amount that 0.4 m of separation is short of the preferred 1 m aisle social distance). We provide the formula for Aisle standing risk as follows:

$$AisleStandingRisk=\sum_{p}\sum_{r\leq RowSit_{p}}\sum_{d^{}}Weight_{d}*RowTime_{{prp}^{\prime }d}$$

where

*p* = passenger advancing towards his/her seat

*r* = row index

*p’* = passenger boarding before passenger *p*

*RowSit*_*p*_ = row in which passenger *p* has a seat

*d* = amount of distance between two closely adjacent aisle passengers

that is less than 1 m

*Weight*_*d*_ = weight to apply when passenger *p’* is *d* m closer to *p* than 1 m.

We set *Weight*_*d*_ to have a value of d.

*RowTime*_*prp’d*_ = time that passenger *p* stands or walks in row *r* while passenger *p’* is

standing in the aisle by *d* m closer to *p* than 1 m

**Individual boarding time** is the time it takes a passenger to board the airplane, that is, the time expressed in seconds between when the passenger enters the airplane through one of the two doors and having completed sitting down. Even though this metric measures the passenger’s boarding time, it may be viewed as a health metric in that higher values of the time spent by a passenger in the aisle might increase his/her health risk due to exposure from other passengers walking or standing in the aisle who may be contagious and potentially have shed the virus prior to a later boarding passenger’s arrival in a particular (potentially infectious) area. This metric has another advantage as an indication of customer satisfaction as passengers prefer their individual boarding times to be short [58].

**Boarding time** is the time in seconds between the moment when the first passenger enters the airplane and the moment when the final passenger to sit, anywhere in the airplane, has taken his or her assigned seat.

### Scenarios

The passengers’ compliance or non-compliance with the prescribed aisle social distance rule of 1 m is modeled by considering various levels of passengers compliance in Table 1.

**Table 1:** Passengers compliance scenarios.

Scenario	Percentages of passengers maintaining the corresponding minimum aisle distance from the passenger directly in front of them			
	0.4 m	0.6 m	0.8 m	1 m
**S1**	0%	0%	0%	100%
**S2**	10%	0%	10%	80%
**S3**	20%	10%	10%	60%
**S4**	30%	10%	10%	50%
**S5**	50%	10%	10%	30%
**S6**	60%	10%	10%	20%
**S7**	80%	0%	10%	10%
**S8**	100%	0%	0%	0%

The first scenario, S_1_, has full compliance in which all the passengers respect the 1 m minimum aisle social distance employed for safety during the pandemic, while the last scenario, S_8_, has no passengers respecting the minimum aisle social distance and thus they follow any passenger in front of them as closely as possible while allowing reasonable space for their personal comfort. We assume that minimum reasonable space for comfort is 0.4 m. In scenario S_2_, 80% of the passengers are compliant, 10% ignore social distancing completely and 10% are nearly compliant respecting 0.8 m aisle social distance. Scenarios S_3_ through S_6_ represent various levels of compliance while scenario S_7_ has only 10% of the passengers in full compliance. As indicated by the values in the table, we conjecture that most passengers are either fully compliant (1 m) or not at all compliant (0.4 m). The other two columns, represent passengers the relatively small percentage of passengers that are nearly compliant (0.8 m) or mostly non-compliant (0.6 m).

### Agent-based modeling

We model the passengers behavior while boarding an airplane through the use of an agent-based approach, implemented in NetLogo 6.2.0 [59]. Selecting this approach has been motivated by the advantages brought by this modeling technique that allows a reasonable representation of the passengers movement and in the given environment. In comparison with a cellular automata approach, the agent-based approach enables the use of various types of agents (called “turtles”) which can move over a given environment. The structure of the environment is similar to the cellular automata approach as the agents called “patches” can possess a series of characteristics which permit building complex forms and structures for representing the environment.

**Fig 8:**
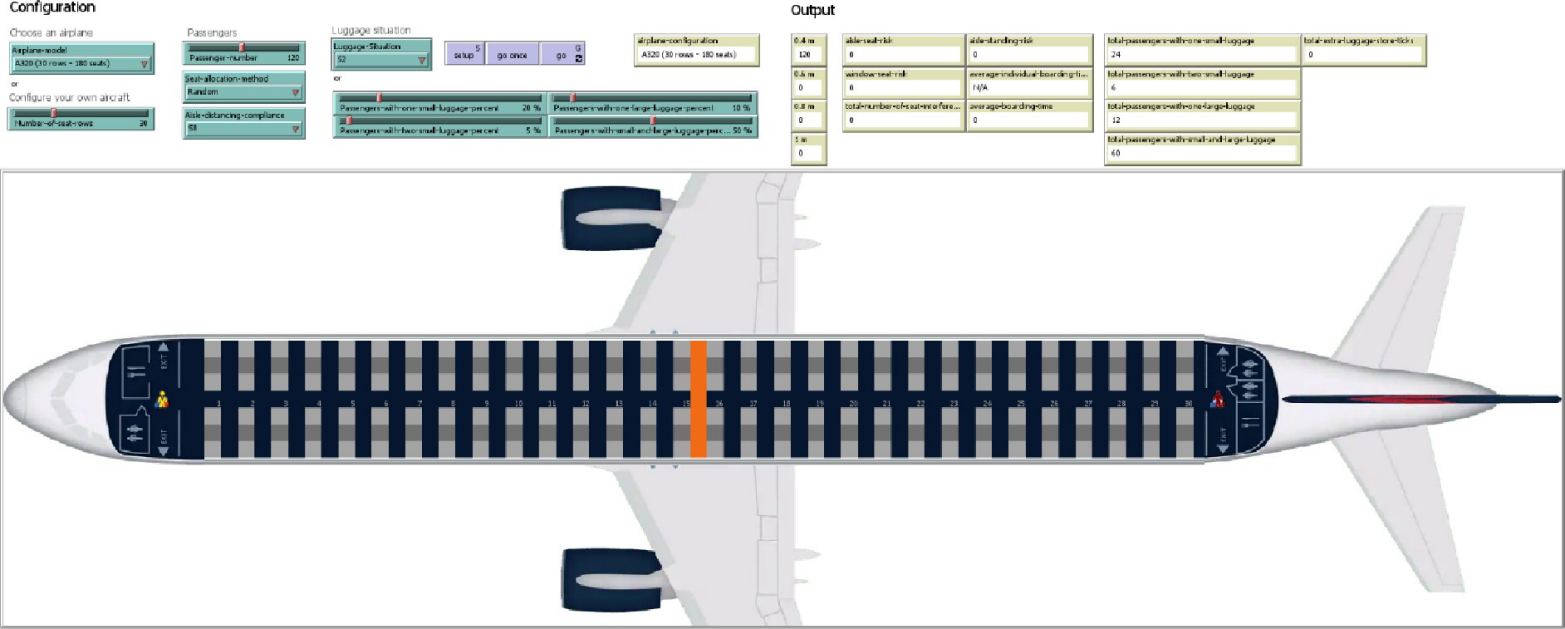
GUI of the agent-based model.

The agent-based graphical user interface (GUI) is depicted in Fig 8. A series of buttons, sliders and switchers have been created in the interface in the “Configuration” section which allows the set-up of the agents’ properties. The real-time values of the risk indicators and operational indicator can be observed in the “Output” section, while the process of passengers boarding is provided through an animated view in the central part of the GUI.

We use two types of agents for modeling the boarding process: patches for creating the environment and turtles for representing the moving passengers. The characteristics of the agents are discussed in the following.

**Fig 9:**
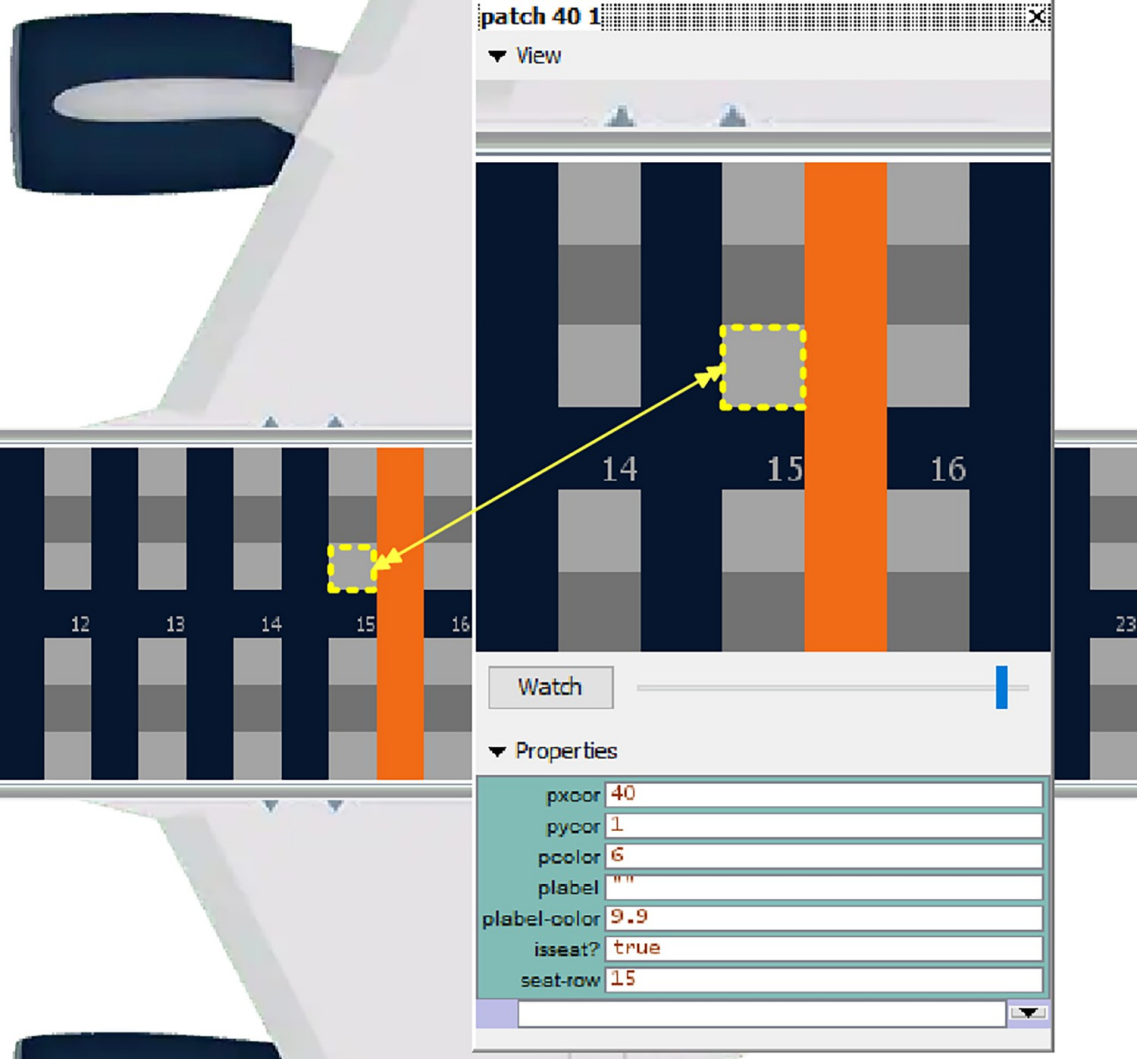
Example of a patch agent.

The patches agents are rectangle parts of the environment, which in our case have been determined based on the scientific literature to correspond to a 0.4 m x 0.4 m area [26, 60, 61], each one of them being positioned at a particular coordinate in the environment, given by the values of the *pxcor* and *pycor* variables. To create the interior of the airplane, different color characteristics have been assigned to the patches through the use of the *pcolor* variable: dark blue for the aisle, light grey for the available seats, dark grey for the middle seat which should be left empty in the pandemic situation, and orange for designating the imaginary middle of the airplane. Two other variables are used: *isseat*? which takes a true value when the patch represents a seat and *seat-row* which indicates the row of seats in which the patch is placed, taking values between 1 and 30, corresponding to the 30 rows of the airplane. Fig 9 presents an example of a patch agent, arbitrarily selected from the model’s environment.

**Fig 10:**
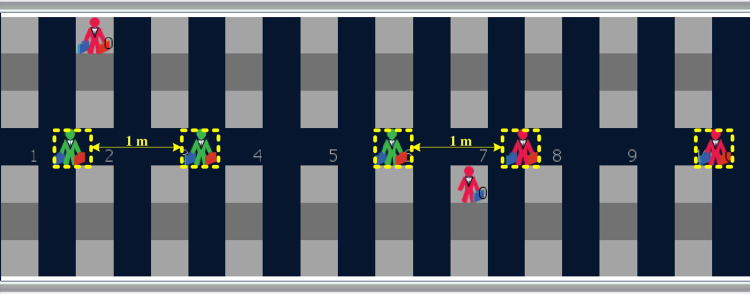
Example of turtle agents.

Turtle agents model the passengers’ actions in the process of airplane boarding and a “person” *shape* has been associated with each of them, having a different *color* corresponding to the boarding group (i.e. apron bus trip) of which they are a member. Turtles move over the patch agents according to a given set of rules and take a position in the environment, characterized by an *xcor* and an *ycor* coordinate. In Fig 10, we observe that the 5 turtle agents advancing in the aisle to their assigned seats have various positions, which do not depend on the patches they are moving over. In this figure, a minimum aisle social distance of 1 m (2.5 patches) is kept between the turtle agents as they advance in the aisle. As noted earlier, some passengers may be noncompliant with the prescribed minimum aisle social distance as determined by the percentages in Table 1.

At the beginning of the simulation, the turtle agents are positioned at the two ends of the airplane and possess a series of characteristics which allows them to replicate the passengers’ movements. The characteristics of the turtle agents are presented in Table 2.

**Table 2:** Turtles characteristics.

Variable	Range / Value	Short description
*speed*	[0, 1]	The speed varies between 0 and 1 patch/tick–the tick is the time unit in NetLogo and it corresponds to 1.2 seconds in real life based on the research literature [61–63]. The agent’s speed is equal to 0 patch/tick when the agent has already occupied its assigned seat or when the agent faces an interference–either an aisle interference, namely the case in which the agent in front of it has stopped in the aisle to take its seat, or to put the luggage into the overhead compartment, or to wait for a type-3 seat interference. Depending on the quantity of luggage carried by an agent, its speed is reduced, ranging between 0.6–0.9 patch/tick. Also, the speed of the agent is reduced if the passenger ahead walks with a slower speed as no passing in the aisle is permitted and the aisle social distance should be preserved to the extent the passenger complies.
*boarding-door*	front / back	Indicates the door through which an agent boards the airplane.
*individual-boarding-start-time*	Z^+^	Retains the moment of time in which each agent has entered the airplane, no matter the door used (back or rear). For the first agents entering through the front and rear doors, this time is equal to zero. For the remaining agents this is the time at which the agent immediately in front of it has proceeded down the aisle by a distance equal to the aisle social distance.
*individual-boarding-duration*	Z^++^	Provides the time needed for each agent to board the airplane. It is a positive integer number and is the time between the *individual-boarding-start-time* and when the individual has sat down.
*luggage*?	true / false	Indicates the presence or the absence of hand luggage with the agent.
*large-luggage*	0 or 1	The number of large luggages carried by the agent.
*small-luggage*	0 or 1	The number of small luggages carried by the agent.
*luggage-store-time*	[0, 6]	The time needed for an agent to store the luggage in the overhead compartment. It is expressed in ticks and it is calculated based on the formula suggested by [64] and used by [62]. The formula takes into account the quantity of luggage stored in the overhead bin by an agent that has previously arrived in the same row and same side of the airplane: Luggage_store_time=((NbinLarge+0.5NbinSmall+NpassengerLarge+0.5NpassengerSmall)*(NpassengerLarge+0.5NPassengerSmall)/2)*Trow Where: *NbinLarge* is the number of large bags in the bin prior to agent’s arrival *NbinSmall* is the number of small bags in the bin prior agent’s arrival *NpassengerLarge* is the number of large bags carried by the agent *NpassengerSmall* is the number of small bags carried by the agent *Trow* is the time for an agent to walk from one row to the next (when not delayed by another agent in front)
*Bus*	1, …, 12	Identifies the apron bus trip to which the agent is allocated according to the boarding scheme used. 1 corresponds to the first bus trip, while 12 to the last bus trip.
*seated*?	true / false	Indicates whether the agent is seated or not.
*agent-seat-row*	1, …, 30	Indicates the seat-row allocated to each agent and they correspond to the 30 rows of the airplane.
*agent-seat-column*	A, C, D, F	Corresponds to the seat columns used by the airlines when assigning a certain seat: A and F are used for the window seats; C and D for the aisle seats. Middle seats are not represented as they are kept empty.
*comfort-distance*	1	Ensures a comfortable personal space between the agents–expressed in number of patches. (1 patch is equivalent to 0.4 m).
*time-to-sit*	1	Time needed for an agent which does not encounter a type-3 seat interference to sit on its assigned seat, expressed in ticks.
*aisle-social-distance*	1, 1.5, 2, 2.5	Expressed in patches, provides the minimum distance to be kept among the agents while advancing in the aisle to their assigned seats. With full compliance, the *aisle-social-distance* is 2.5 patches (equivalent to 1 m–Fig 10), while in a perfect non-compliance case, it is equal to the *comfort-distance* of 1 patch.

As the simulation starts, the agents allocated to the first bus, based on the airplane boarding scheme, proceed to their assigned seats, each of them possessing its own set of characteristics. While walking down the aisle the agents keep a distance equal to at least the agent’s *aisle-social-distance* from the agent ahead. The distance between the agents can be higher than the *aisle-social-distance* when the agent ahead has a faster speed, as a result of its own characteristics or as a result of a smaller number of hand luggage. The passengers’ movements are subject to the restriction that a given position can be only occupied by a turtle agent [65], this constraint being a result of the particular value for the *aisle-social-distance*. Each simulation trial assumes that 15% of the agents travel with no hand luggage, 20% with one small bag, 5% with two small bags, 10% with one large bag and the remaining 50% with one small and one large bag. Each simulation trial applies this frequency of luggage percentages, but the individual passengers carrying a particular combination of hand luggage are chosen at random. Similarly, each simulation trial assumes the number of passengers with a particular *aisle-social-distance* corresponds exactly and deterministically to the frequency percentages of Table 1, but that the individual passengers having the particular value of the *aisle-social-distance* is chosen at random.

We assume that none of the passengers assigned to a particular apron bus will choose another bus by mistake and that the flow of passengers through the two airplane doors is continuous, meaning that there is no time between the arrival to the boarding door between the last passenger of a group and the first passenger of the subsequent group.

Upon arriving near its assigned seat, the agent places its carry-on luggage (if any) in the overhead bin compartment and blocks the aisle a time equal to *luggage-store-time*. If the agent has the seat near the window and the agent with aisle seat in the same row and in the same side of the aisle has arrived to its assigned seat, the agent will be involved in a type-3 seat interference and will block the aisle for another 8–11 ticks [61], the specific time generated at random according to the uniform distribution. If no type-3 seat interference is experienced by the agent, it will block the aisle for 1 tick, representing the *time-to-sit*.

The simulation ends when the last agent takes its assigned seat.

## Simulation results

Each the 6 methods for airplane boarding when 12 apron bus trips are used for the passengers’ transport from the airport to the airplane are simulated 10,000 times for each of the 8 aisle social distance compliance scenarios. The performances of the methods are compared to a baseline scenario represented by the Random boarding method in which 10 passengers are assigned at random to each apron bus.

A total of 560,000 simulations have been performed through the BehaviourSpace tool provided by NetLogo [66] and the average values for the performance indicators are determined and discussed in the following.

### Numerical results for aisle seat risk

The aisle seat risk measures the potential risk of contagion for the passengers already seated in the aisle seats while the potentially contagious passengers walk in the aisle towards their assigned seats, as they might spread the coronavirus to the passengers they are passing by. The risk is measured in seconds and the values obtained for the 6 boarding methods under investigation, plus the baseline Random boarding method are presented in Table 3. The best (lowest) duration for each compliance scenario is highlighted in **bold** face type.

**Table 3:** Aisle seat risk.

Boarding method	Aisle seat risk							
	S_1_	S_2_	S_3_	S_4_	S_5_	S_6_	S_7_	S_8_
Baseline: Random	4522	4537	4546	4563	4618	4655	4691	4731
Back-to-front	667	669	671	670	672	674	675	676
WilMA–Back-to-front	349	353	357	359	364	366	369	370
WilMA–Spread	1478	1478	1478	1479	1481	1482	1486	1493
WilMA–Back-to-front–one-per-row	629	633	636	637	643	645	646	647
Reverse-pyramid–Steep	**234**	**235**	**237**	**237**	**239**	**240**	**241**	**241**
Reverse-pyramid–Spread	**234**	**235**	**237**	**237**	**239**	**240**	**241**	**241**

**Fig 11:**
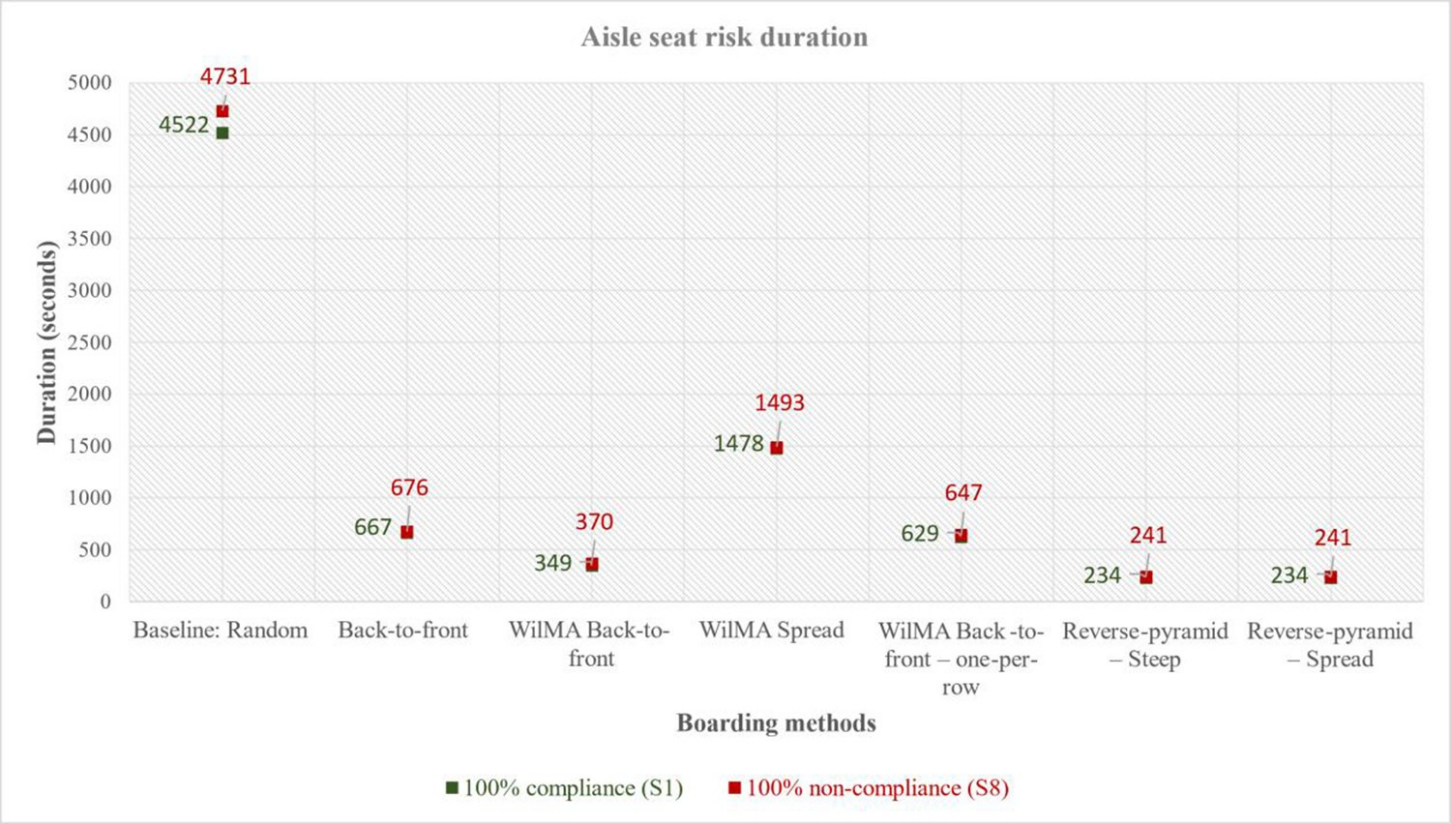
Comparison of aisle seat risk duration for the boarding methods in S_1_ versus S_8_.

From Table 3, we observe that this risk increases for each of the boarding methods when the compliance with the prescribed social distance decreases from scenario S_1_ to S_8_. This relationship is consistent with [5] which provides insight into why aisle seat risk is lower when the distance in the aisle between passengers is higher. Reducing compliance is analogous to shortening the distance between passengers in the aisle in the earlier work. Overall, the differences in aisle risk between the S_1_ scenario (characterized by 100% compliance with keeping the 1 m aisle social distance) and S_8_ scenario (characterized by no compliance) range between 1.35% (in the case of Back-to-front) and 6.02% (for WilMA–Back-to-front). Fig 11 presents the comparison between the S_1_ and S_8_ scenarios for all the airplane boarding methods.

The aisle seat risk is lower in the case of Reverse-pyramid–Steep and Reverse-pyramid–Spread than for the other boarding methods. Compared to the Random boarding method, the aisle seat risk for the two methods (Reverse-pyramid–Steep and Reverse-pyramid–Spread) is 94.79% - 94.91% lower–depending on the degree of compliance.

The fourth and fifth best results for aisle seat risk are provided by WilMA–Back-to-front–one-per-row and Back-to-front. The aisle seat risk is reduced by up to 86.32% in the case of WilMA–Back-to-front–one-per-row and by up to 85.71% in the case of Back-to-front when compared to Random boarding. The worst of the six methods in terms of aisle seat risk is WilMA–Spread, which reduces the risk by up to 68.44% when compared to Random boarding.

### Numerical results for window seat risk

The window seat risk measures the total duration of time in which window seat passengers might contact the disease from potentially contagious passengers as they walk down the aisle to their assigned seats. Compared to the aisle seat risk, the window seat risk is less important as the distance between the passenger walking the aisle and the potentially affected seated passenger is greater in the case of window seats than in the case of aisle seats.

The average values obtained for the window seat risk through the simulations are presented in Table 4. As with the analysis of aisle seat risk, the window seat risk increases for each of the boarding methods when the compliance with the prescribed aisle social distance decreases. When the compliance decreases from S_1_ (100% compliance) to S_8_ (no compliance), the window seat risk increases between 0.93% (for Reverse pyramid–Steep) and 6.75% (for Back-to-front).

**Table 4:** Window seat risk.

Boarding method	Window seat risk							
	S_1_	S_2_	S_3_	S_4_	S_5_	S_6_	S_7_	S_8_
Baseline: Random	4201	4216	4259	4281	4336	4361	4399	4439
Back-to-front	**919**	**931**	**943**	**950**	**963**	**971**	**978**	**981**
WilMA–Back-to-front	4892	4918	4950	4956	4973	4974	4980	4995
WilMA–Spread	5137	5150	5166	5180	5209	5225	5249	5280
WilMA–Back-to-front–one-per-row	2169	2179	2190	2195	2208	2213	2214	2215
Reverse-pyramid–Steep	2156	2164	2172	2175	2175	2175	2176	2176
Reverse-pyramid–Spread	2361	2369	2381	2384	2390	2390	2391	2396

The highest values of window seat risk result from the WilMA–Spread method and the second highest values from the WilMA–Back-to-front boarding method. For both of these methods, the window seat passengers are composed entirely of passengers from the first six apron bus trips. This results in these passengers being seated while passengers from the remaining six apron bus trips walk past them. Consequently, it is not surprising that these two methods result in higher window seat risk than the other boarding methods as the latter methods have window seat passengers from 10 to 12 bus trips depending on the method. The WilMA–Spread method has its window seat passengers of an average bus trip spread out more across the airplane rows than the WilMA–Back-to-front method which tends to assign each subsequent trip’s window seat passengers closer towards the back. This additional spreading of WilMA–Spread’s window seat passengers leads to more of them being passed by later boarding passengers than WilMA–Back-to-front and thus leading to higher window seat risk.

The Random boarding method will often result in each bus trip containing window seat passengers, which is favorable for window seat risk. However, the passenger with a particular window seat is equally likely to be assigned to any apron bus. This contrasts with the other methods that all tend to assign passengers having window seats closer to the back to the earlier apron buses, on average. Those two factors—one favorable for window seat risk and the other unfavorable—results in the Random boarding method providing the third highest window seat risk.

The lowest window seat risk results from the Back-to-front method. With this method, the window seat passengers are passed by only a small number of later boarding passengers.

The remaining three boarding methods have similar amounts of window seat risk. Reverse-pyramid—Steep has less window seat risk than Reverse-pyramid–Spread due to the different degrees of spreading the window seat passengers. Meanwhile, the window seat passengers of WilMA–Back-to-front method have more spreading than Reverse-pyramid–Steep and less spreading than Reverse-pyramid–Spread resulting in its values of window seat risk being between those of the two Reverse-pyramid methods.

### Numerical results for total number of seat interferences

The total number of seat interferences are shown in Table 5. The number of seat interferences is independent of the level of compliance of the passengers in keeping the prescribed aisle social distance of 1 m.

**Table 5:** Total number of seat interferences.

Boarding method	Total number of seat interferences							
	S_1_	S_2_	S_3_	S_4_	S_5_	S_6_	S_7_	S_8_
Baseline: Random	30	30	30	30	30	30	30	30
Back-to-front	18	18	18	18	18	18	18	18
WilMA–Back-to-front	**0**	**0**	**0**	**0**	**0**	**0**	**0**	**0**
WilMA–Spread	**0**	**0**	**0**	**0**	**0**	**0**	**0**	**0**
WilMA–Back-to-front–one-per-row	**0**	**0**	**0**	**0**	**0**	**0**	**0**	**0**
Reverse-pyramid–Steep	**0**	**0**	**0**	**0**	**0**	**0**	**0**	**0**
Reverse-pyramid–Spread	**0**	**0**	**0**	**0**	**0**	**0**	**0**	**0**

For the methods involving WilMA and Reverse-pyramid, there are no seat interferences as each window seat passenger takes an earlier bus than the passenger seated in an aisle seat of the same row. For the Random boarding method, 50% of the time an aisle seat passenger will sit prior to the window seat passenger of the same row and side of the airplane. With 60 aisle seat passengers, this results in 30 seat interferences with Random boarding as indicated in the table.

With the Back-to-front method, there are four aisle seat passengers in the first apron bus who may arrive at their seats earlier or later than the four window seat passengers sitting in their rows, which are rows 15 and 16. With the method, there are two aisle seat passengers in the second apron bus who may arrive at their seats earlier than the two window seat passengers sitting in their rows, which are rows 14 and 17. In total, there are 36 aisle seat passengers with the Back-to-front method who may board prior to the window seat passengers sitting in their rows. With a 50% probability of a seat interference in each case, the 18 (= 36 * 0.5) average number of seat interferences in Table 5 for the Back-to-front method make sense.

### Numerical results for aisle standing risk

Table 6 shows the aisle standing risk as a function of the boarding methods and level of compliance with the prescribed 1 m aisle social distance. This risk is zero for all the methods in the S_1_ scenario as in this case all the passengers respect the prescribed social distance. As the level of compliance decreases, the value of this health risk increases. The risk increases by 9.2 to 10.2 times (depending on the boarding method) when comparing a high compliance scenario S_2_ (in which 80% of the passengers keep a 1 m distance from the passenger ahead) with a non-compliance scenario S_8_ (in which none of the passengers respect the 1 m aisle social distance).

**Table 6:** Aisle standing risk.

Boarding method	Aisle standing risk							
	S_1_	S_2_	S_3_	S_4_	S_5_	S_6_	S_7_	S_8_
Baseline: Random	0	50	103	156	266	324	438	561
Back-to-front	0	91	186	282	479	580	772	954
WilMA–Back-to-front	0	69	139	210	354	426	566	703
WilMA–Spread	**0**	**38**	**76**	**116**	**196**	**238**	**322**	**412**
WilMA–Back-to-front–one-per-row	0	63	127	193	326	394	530	667
Reverse-pyramid–Steep	0	59	120	181	306	371	499	630
Reverse-pyramid–Spread	0	56	112	170	287	347	465	585

The methods that have a greater number of rows of separation between passengers of a same airplane boarding group (e.g. more spread) tend to have lower values of aisle standing risk than those methods that are more likely to result in congestion from passengers from the same apron bus trip having seats in close proximity of each other. For example, the method with the most spread, WilMA–Spread, has the lowest values of this risk while the method with the most congestion, Back-to-front has the highest values. We see the same pattern for the other methods, for instance, that Reverse-pyramid–Spread has lower risk than Reverse-pyramid–Steep.

### Numerical results for individual boarding time

Many of the same patterns observed in Table 6 for aisle standing risk repeat themselves in Table 7 that contains the individual boarding time. For each boarding method, when the level of compliance with the aisle social distance decreases, the individual boarding time increases. WilMA–Spread has the lowest average individual boarding time for each level of compliance and Back-to-front the worst. The other boarding methods tend to have the same ordinal performance, namely, the more spread and less congestion of the method, the better the value of individual boarding time. One exception is that the Random method provides the second-best performance for aisle standing risk but only the sixth best performance for individual boarding time. The reason for the Random method’s discrepancy in its relative performance is that its individual boarding times are impacted by its high number of seat interferences by more than its aisle standing risks are impacted. If we consider the ordinal ranking within those methods that board window seat passengers before middle seat passengers in the same row and same side of the airplane, namely the Wilma and Reverse-pyramid methods, the ordinal ranking of these methods is the same for aisle standing risk as it is for individual boarding time. Thus, individual boarding time is a good proxy for comparing the ordinal position of these methods (i.e. which methods are better than the others).

**Table 7:** Individual boarding time.

Boarding method	Individual boarding time							
	S_1_	S_2_	S_3_	S_4_	S_5_	S_6_	S_7_	S_8_
Baseline: Random	39	39	40	40	40	40	41	41
Back-to-front	48	48	49	49	50	50	50	50
WilMA–Back-to-front	39	39	39	39	39	39	39	39
WilMA–Spread	**33**	**33**	**33**	**33**	**33**	**34**	**34**	**34**
WilMA–Back-to-front–one-per-row	37	37	38	38	38	38	38	38
Reverse-pyramid–Steep	36	36	37	37	37	37	37	37
Reverse-pyramid–Spread	35	36	36	36	36	36	36	36

The absolute differences in average individual boarding times between the methods and levels of compliance are much smaller than the absolute differences among the aisle standing risk. For instance, the Back-to-front method with no compliance (S_8_) has 18 times as much aisle standing risk as the WilMA–Back-to-front method with high compliance (S_2_) but only 28% more individual boarding time. Consequently, the metric of individual boarding time may be a good proxy for aisle standing risk for the purposes of comparing the ordinal performance of alternative boarding methods that board a row and side’s window seat passengers before its middle seat passengers but a bad proxy for the purposes of comparing the magnitude of those differences in aisle standing risk. Because individual boarding time is simple to calculate, easy to understand, and provides an indication of passenger satisfaction, it may be the superior metric to use with the understanding that it provides only a relative (and not an absolute) basis in comparing the aisle standing risk of alternative boarding methods that incur no seat interferences.

### Numerical results for boarding time

As expected, the boarding time decreases for all the airplane boarding methods as the compliance of the passengers to the 1 m aisle social distance rule decreases–Table 8. The average boarding time is reduced between 31.35% (Random) and 40.45% (WilMA–Back-to-front) when the level of aisle social distance compliance varies from 100% (S_1_) to 0% (S_8_).

**Table 8:** Boarding time.

Boarding method	Boarding time							
	S_1_	S_2_	S_3_	S_4_	S_5_	S_6_	S_7_	S_8_
Baseline: Random	555	530	503	487	454	437	412	381
Back-to-front	597	573	541	519	474	451	414	364
WilMA–Back-to-front	492	469	439	420	381	361	332	293
WilMA–Spread	464	444	418	403	372	356	335	307
WilMA–Back-to-front–one-per-row	477	455	427	409	374	356	330	295
Reverse-pyramid–Steep	459	437	409	392	358	340	315	283
Reverse-pyramid–Spread	**451**	**430**	**402**	**385**	**350**	**332**	**308**	**276**

For this metric, the best boarding method is Reverse-pyramid–Spread followed closely by Reverse-pyramid–Steep. This relationship is consistent with an earlier work involving ten apron bus trips [5]. The highest (worst) boarding time results from the Back-to-front method followed by Random boarding.

Of the WilMA methods, WilMA–Spread is best for all but the lowest levels of compliance. For low compliance scenario S_7_, WilMA–Back-to-front–one-per-row is the best of the three and WilMA–Back-to-front also outperforms WilMA–Spread. For the no compliance scenario S_8_, WilMA–Back-to-front is the best of the WilMA methods followed by WilMA–Back-to-front–one-per-row.

## Discussion

Table 9 indicates the relative performance of the 7 airplane boarding methods for each health risk indicator and for the operational indicator represented by the boarding time based on the performance results averaged across all compliance scenarios. For methods that are neither best nor worst for a particular indicator, their position in the table is based on their approximate average performance compared with the best and worst methods. For example, when evaluating aisle seat risk, even though WilMA–Spread is the 6^th^ best method, its average performance is closer to that of the best performing methods than it is to the worst performing method and thus WilMA–Spread is positioned in the left half of the table for aisle seat risk.

**Table 9:** Airplane boarding methods comparison based on the performance indicators.

Indicator	Airplane boarding method		
	Best	to	Worst
**Aisle seat risk**	1. Reverse-pyramid–Steep		
	1. Reverse-pyramid–Spread		
	2. WilMA–Back-to-front		
	3. WilMA–Back-to-front–one-per-row		
	4. Back-to-front		
	5. WilMA–Spread		
	6. Random		
**Window seat risk**	1. Back-to-front		
	2. Reverse-pyramid–Steep		
	3. WilMA–Back-to-front–one-per-row		
	4. Reverse-pyramid–Spread		
	5. Random		
	6. WilMA–Back-to-front		
	7. WilMA–Spread		
**Total number of seat interferences**	1. Reverse-pyramid–Steep		
	1. Reverse-pyramid–Spread		
	1. WilMA–Back-to-front		
	1. WilMA–Spread		
	1. WilMA–Back-to-front–one-per-row		
	2. Back-to-front		
	3. Random		
**Aisle standing risk**	1. WilMA Spread		
	2. Random		
	3. Reverse-pyramid–Spread		
	4. Reverse-pyramid–Steep		
	5. WilMA–Back-to-front–one-per-row		
	6. WilMA–Back-to-front		
	7. Back-to-front		
**Individual boarding time**	1. WilMA–Spread		
	2. Reverse-pyramid–Spread		
	3. Reverse-pyramid–Steep		
	4. WilMA–Back-to-front–one-per-row		
	5. WilMA–Back-to-front		
	6. Random		
	7. Back-to-front		
**Boarding time**	1. Reverse-pyramid–Spread		
	2. Reverse-pyramid–Steep		
	3. WilMA–Spread		
	4. WilMA–Back-to-front–one-per-row		
	5. WilMA–Back-to-front		
	6. Random		
	7. Back-to-front		

The Back-to-front method has the best values of window seat risk. However, when the other metrics are considered, Back-to-front performs poorly enough that it is difficult to imagine an airline preferring it over another method. Furthermore, the window seat risk is less important than aisle seat risk (because window seat passengers are further from the aisle than aisle seat passengers) and we suspect the other health metrics as well. Similarly, the Random method has the second-best performance for aisle standing risk but does poorly on the other metrics.

If aisle standing risk is the most important consideration, then the WilMA–Spread method should be used. In addition to minimizing aisle standing risk, WilMA–Spread has the best values of individual boarding time and yields zero seat interferences. WilMA–Spread has the third best performance for average boarding time.

The best performers for average boarding time are Reverse-pyramid–Spread and Reverse-pyramid–Steep. These methods have the lowest aisle seat risk, have lower window seat risk than WilMA–Spread, and trail only WilMA–Spread for aisle standing risk. They are good candidates for airlines to consider unless they prefer WilMA–Spread’s advantage in aisle standing risk and individual boarding time. Reverse-pyramid–Spread has slightly better boarding times, aisle standing risk, and individual boarding time than Reverse-pyramid–Steep, but with worse window seat risk.

Table 10 illustrates the percentage changes in four of the performance metrics when comparing the full compliance scenario (S_8_) with the no compliance scenario (S_1_), for each of the 7 boarding methods. We observe that Reverse-pyramid–Steep is slightly less affected than Reverse-pyramid–Spread by changes in the passengers’ compliance to the 1 m aisle social distance rule. WilMA–Spread results in less churn as the level of compliance changes than Reverse-pyramid Steep for aisle seat risk and boarding time but more churn for window seat risk and individual boarding time.

**Table 10:** Changes in the selected indicators in non-compliance scenario (S_8_) compared to compliance scenario (S_1_).

Boarding method	% of change in the indicator in S_8_ compared to S_1_			
	Aisle seat risk	Window seat risk	Individual boarding time	Boarding time
Baseline: Random	4.62%	5.67%	5.13%	-31.35%
Back-to-front	1.35%	6.75%	4.17%	-39.03%
WilMA–Back-to-front	6.02%	2.11%	0.00%	-40.45%
WilMA–Spread	1.01%	2.78%	3.03%	-33.84%
WilMA–Back-to-front–one-per-row	2.86%	2.12%	2.70%	-38.16%
Reverse-pyramid–Steep	2.99%	0.93%	2.78%	-38.34%
Reverse-pyramid–Spread	2.99%	1.48%	2.86%	-38.80%

## Conclusions

In this paper, we consider social distancing measures recommended during a pandemic for reducing the risk of passengers contracting the disease. These measures reduce the capacity of apron buses transporting the passengers from the airport terminal to the airplane, and the capacity of the airplane by keeping the middle seat empty. Furthermore, the airline (or the government) prescribes a 1 m minimum social distance to be kept between passengers walking down the aisle or standing in the aisle. However, there may be passengers who do not comply with the prescribed aisle social distance.

We analyze the performance of six airplane boarding methods adapted for this condition, while varying the level of passenger compliance with the prescribed aisle social distance between full compliance and no compliance. We measure the performance in terms of health risk to passengers and the time needed to complete the boarding of the airplane. The health metrics pertain to the number of seat interferences, the risk to seated passengers from later boarding passengers walking past them, and *aisle standing risk*. This latter metric is a weighted duration of the time all passengers spend walking or waiting in the aisle when they are closer than 1 m to any previously boarded passenger in front of them in the aisle. The weights are larger when the distance between passengers in the aisle is smaller.

Through the agent-based simulations, we observe that the Back-to-front and Random boarding methods perform poorly. We test three WilMA methods in which the best one, WilMA–Spread, assigns passengers to apron bus trips so that the passengers in each bus trip sit as far apart from each other as possible while honoring the WilMA principle that all window seat passengers board before any aisle seat passengers. WilMA–Spread results in the lowest values of all methods for aisle standing risk. It also results in the best (lowest) values of individual boarding time. We find that the average individual boarding time is a good proxy for ranking the performance of boarding methods in which the row and side of an airplane’s window seat passengers board before its aisle seat passengers. Also, average individual boarding time is easier to understand, simpler to calculate, and better expresses the customer satisfaction due to passengers spending less time progressing to their seats. Furthermore, WilMA–Spread results in zero seat interferences and the provides the third shortest time to complete boarding of the entire airplane.

Reverse-pyramid–Spread has the shortest time to complete boarding of the airplane. It also has zero seat interferences and the lowest risk to passengers seated in the aisle from later boarding passengers walking past them. This method and WilMA–Spread should both be seriously considered by airlines who are interested in passenger health, passenger satisfaction, and the time to complete boarding of the airplane. Reverse-pyramid–Steep performs as well (or nearly as well) as Reverse-pyramid–Spread for the metrics, except for the risk to window seat passengers from later boarding passengers, in which it performs slightly better. This latter metric does not appear as important as the other metrics for which Reverse-pyramid–Spread performs better.

The passengers’ non-compliance with aisle social distance can adversely impact the health metric of a boarding method by up to 6.75%—depending on the boarding method and the performance metric. In addition to hindering aisle standing risk, non-compliance to the aisle social distance increases the health risk to seated passengers from later boarding passengers during the boarding process. However, non-compliance improves (decreases) the time to complete boarding of the airplane, by up to 38.8%, even though it worsens (lengthens) the average time an individual spends boarding.

Future research can be conducted to consider passengers’ non-compliance with other rules such as arriving late at the boarding gate. Such delays can result from personal reasons of the individual passengers (e.g. late to depart for the airport, driving path taken to the airport) or due to factors at the airport largely beyond their control (e.g. passport control, luggage scanning, and checked luggage). A robustness check for the airplane boarding methods can be performed to evaluate the impact of the perturbations in the boarding process [67]. Further research could analyze the impacts of passenger compliance when alternative airplane configurations are used (e.g. wide body airplane) and the use of jet bridges instead of apron buses.

The paper is accompanied by a series of videos made for the 7 airplane boarding methods (including Random) with the S_4_ aisle social distance compliance scenario. The videos can be accessed at the following link: https://github.com/liviucotfas/plosone-covid19-apron

## Supporting information

S1 File Video recording of Baseline: Random. (GIF)

S2 File Video recording of Back-to-front. (GIF)

S3 File Video recording of WilMA–Back-to-front. (GIF)

S4 File Video recording of WilMA–Spread. (GIF)

S5 File Video recording of WilMA–Back-to-front–one-per-row. (GIF)

S6 File Video recording of Reverse-pyramid–Steep. (GIF)

S7 File Video recording of Reverse-pyramid–Spread. (GIF)

S1 Graphical abstract (DOCX)
