# Supplementary material for: Health risks of airplane boarding methods with apron buses when some passengers disregard safe social distancing
Source: PLoS One. 2022 Aug 1;17(8):e0271544. doi: 10.1371/journal.pone.0271544 (PMC9342771; doi:10.1371/journal.pone.0271544)
Supplement: S3 File — (GIF) [file pone.0271544.s003.gif]

safety-reliability-transportation-systems-covid19-apron/wilma-btf.gif at main · liviucotfas/safety-reliability-transportation-systems-covid19-apron · GitHub


Skip to content


Sign up

- Why GitHub?

  - Features →
  - Mobile →
  - Actions →
  - Codespaces →
  - Packages →
  - Security →
  - Code review →
  - Issues →
  - Integrations →
  - GitHub Sponsors →
  - Customer stories →
- Team
- Enterprise
- Explore

  - Explore GitHub →
  - Learn and contribute
  - Topics →
  - Collections →
  - Trending →
  - Learning Lab →
  - Open source guides →
  - Connect with others
  - The ReadME Project →
  - Events →
  - Community forum →
  - GitHub Education →
  - GitHub Stars program →
- Marketplace
- Pricing

  - Plans →
  - Compare plans →
  - Contact Sales →
  - Education →

- In this repository

  All GitHub
  ↵

  Jump to
  ↵

- No suggested jump to results

- In this repository

  All GitHub
  ↵

  Jump to
  ↵
- In this user

  All GitHub
  ↵

  Jump to
  ↵
- In this repository

  All GitHub
  ↵

  Jump to
  ↵

Sign in

Sign up

{{ message }}

# liviucotfas / **safety-reliability-transportation-systems-covid19-apron** Public

- Notifications
- Star
   
  0
- Fork

  0

- Code
- Issues
  0
- Pull requests
  0
- Actions
- Projects
  0
- Wiki
- Security
- Insights

More

- Code
- Issues
- Pull requests
- Actions
- Projects
- Wiki
- Security
- Insights

Permalink

main

Switch branches/tags


Branches
Tags

Could not load branches


Nothing to show


Loading

{{ refName }}
default
View all branches

Could not load tags


Nothing to show


{{ refName }}
default

Loading

View all tags

## safety-reliability-transportation-systems-covid19-apron/recordings/**wilma-btf.gif**

Go to file


- Go to file
  T
- Go to line
  L
- Copy path
- Copy permalink

Cannot retrieve contributors at this time

2.48 MB

Download

- Open with Desktop
- Download

Go

- © 2021 GitHub, Inc.
- Terms
- Privacy
- Security
- Status
- Docs


- Contact GitHub
- Pricing
- API
- Training
- Blog
- About

You can’t perform that action at this time.

You signed in with another tab or window. Reload to refresh your session.
You signed out in another tab or window. Reload to refresh your session.
