# Supplementary figures and images for: Health risks of airplane boarding methods with apron buses when some passengers disregard safe social distancing
Source: PLoS One. 2022 Aug 1;17(8):e0271544. doi: 10.1371/journal.pone.0271544 (PMC9342771; doi:10.1371/journal.pone.0271544)

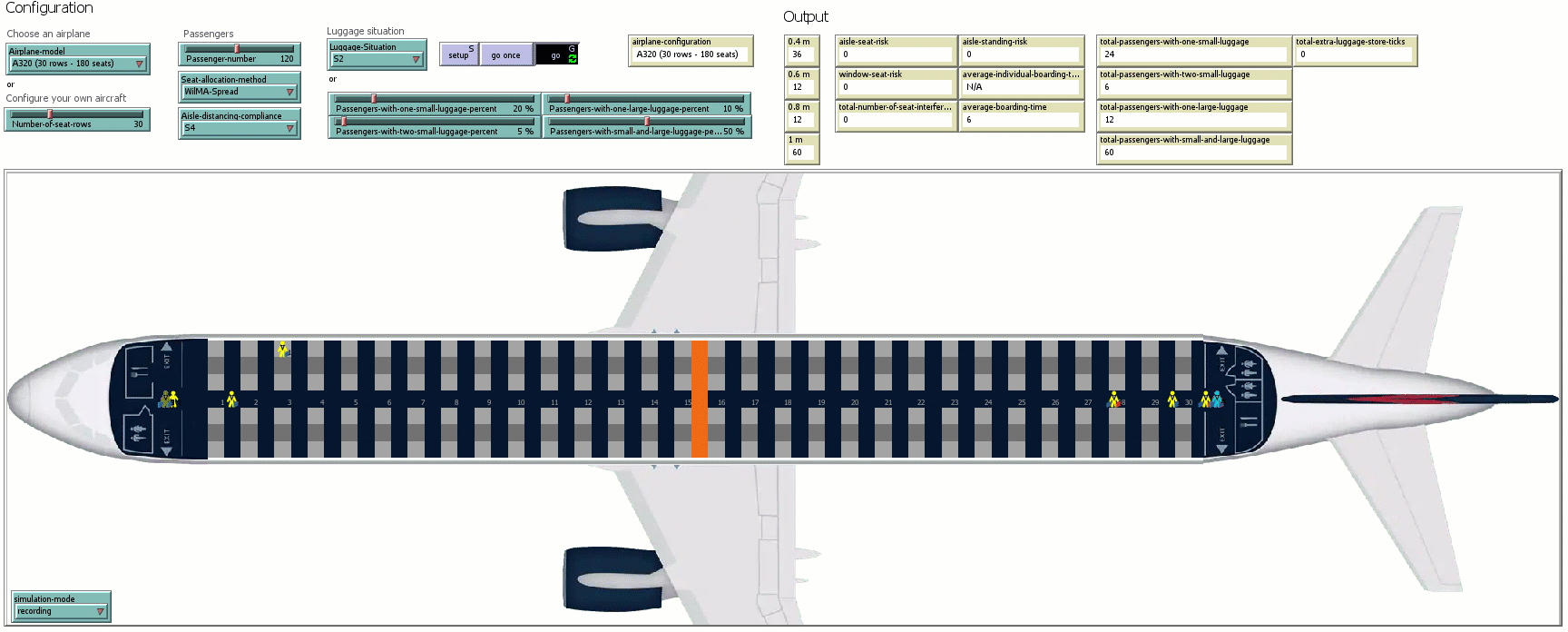

Supplement: S4 File — (GIF) [file pone.0271544.s004.gif]
