# Supplementary material for: Health risks of airplane boarding methods with apron buses when some passengers disregard safe social distancing
Source: PLoS One. 2022 Aug 1;17(8):e0271544. doi: 10.1371/journal.pone.0271544 (PMC9342771; doi:10.1371/journal.pone.0271544)
Supplement: S1 Graphical abstract — (DOCX) [file pone.0271544.s008.docx]

Graphical abstract:
